# Acetazolamide and human carbonic anhydrases: retrospect, review and discussion of an intimate relationship

**DOI:** 10.1080/14756366.2023.2291336

**Published:** 2023-12-11

**Authors:** Dimitrios Tsikas

**Affiliations:** Core Unit Proteomics, Institute of Toxicology, Hannover Medical School, Hannover, Germany

**Keywords:** Acetazolamide, amino acids, bicarbonate, carbonic anhydrases, excretion, inhibition, mechanisms, tolerance, transporters

## Abstract

Acetazolamide (AZM) is a strong pharmacological sulphonamide-type (R-SO_2_-NH_2_, p*K*_a_ 7.2) inhibitor of the activity of several carbonic anhydrase (CA) isoforms, notably of renal CA II (*K*_i_, 12 nM) and CA IV (*K*_i_, 74 nM). AZM is clinically used for about eighty years in various diseases including epilepsy and glaucoma. Pharmacological AZM increases temporarily the urinary excretion of bicarbonate (HCO_3_^–^) and sodium ions (Na^+^) and sustainably the urinary pH. AZM is excreted almost unchanged over several hours at high rates in the urine. Closely parallel concentrations of circulating and excretory AZM are observed upon administration of therapeutical doses of AZM. In a proof-of-principle study, we investigated the effects of the ingestion of a 250-mg AZM-containing tablet by a healthy volunteer on the urinary excretion of organic and inorganic substances over 5 h (range, 0, 0.5, 1, 1.5, 2, 3, 4, 5 h). Measured analytes included: AZM, amino acids and their metabolites such as guanidinoacetate, i.e. the precursor of creatine, of asymmetrically (ADMA) and symmetrically (SDMA) dimethylated arginine, nitrite (O = N-O^–^, p*K*_a_ 3.4) and nitrate (O_2_N-O^–^, p*K*_a_ −1.37), the major metabolites of nitric oxide (NO), the C-H acidic malondialdehyde (MDA; (CHO)_2_CH_2_, p*K*_a_ 4.5), and creatinine for correction of analytes excretion. All analytes were measured by validated isotopologues using gas chromatography-mass spectrometry (GC-MS) methods. AZM excretion in the urine reached its maximum value after 2 h and was fairly stable for the next 3 h. Time series analysis by the ARIMA method was performed. AZM ingestion increased temporarily the urinary excretion of the amino acids Leu + Ile, nitrite and nitrate, decreased temporarily the urinary excretion of other amino acids. AZM decreased sustainably the urinary excretion of MDA, a biomarker of oxidative stress (i.e. lipid peroxidation). Whether this decrease is due to inhibition of the excretion of MDA or attenuation of oxidative stress by AZM is unknown. The acute and chronic effects of AZM on the urinary excretion of electrolytes and physiological substances reported in the literature are discussed in depth in the light of its extraordinary pharmacokinetics and pharmacodynamics. Tolerance development/drug resistance to AZM in chronic use and potential mechanisms are also addressed.

## Acetazolamide and carbonic anhydrases – general aspects

Carbonic anhydrases (CA; EC 4.2.1.1) are ubiquitous and abundant metalloenzymes. They catalyse the conversion of CO_2_ to bicarbonate (HCO_3_^–^) and the protonation of bicarbonate to CO_2_ and H_2_O (CO_2_ + H_2_O ↔ HCO_3_^–^ + H^+^) by several orders of magnitude. Carbonic acid (H_2_CO_3_) is considered the intermediate reaction product of CA-catalysed hydration of CO_2_. H_2_CO_3_ is extremely labile in aqueous systems under ambient conditions of temperature and pressure and has not been isolated thus far^1^. Recently, the p*K*_a_ value of H_2_CO_3_ is estimated to be 3.45: H_2_CO_3_ ↔ HCO_3_^–^ + H^+^^2^. This value is substantially lower than the p*K*_a_ value of 6.35 that is commonly assumed on the basis of the overall CO_2_/HCO_3_^–^ equilibrium.

**Figure 1.**
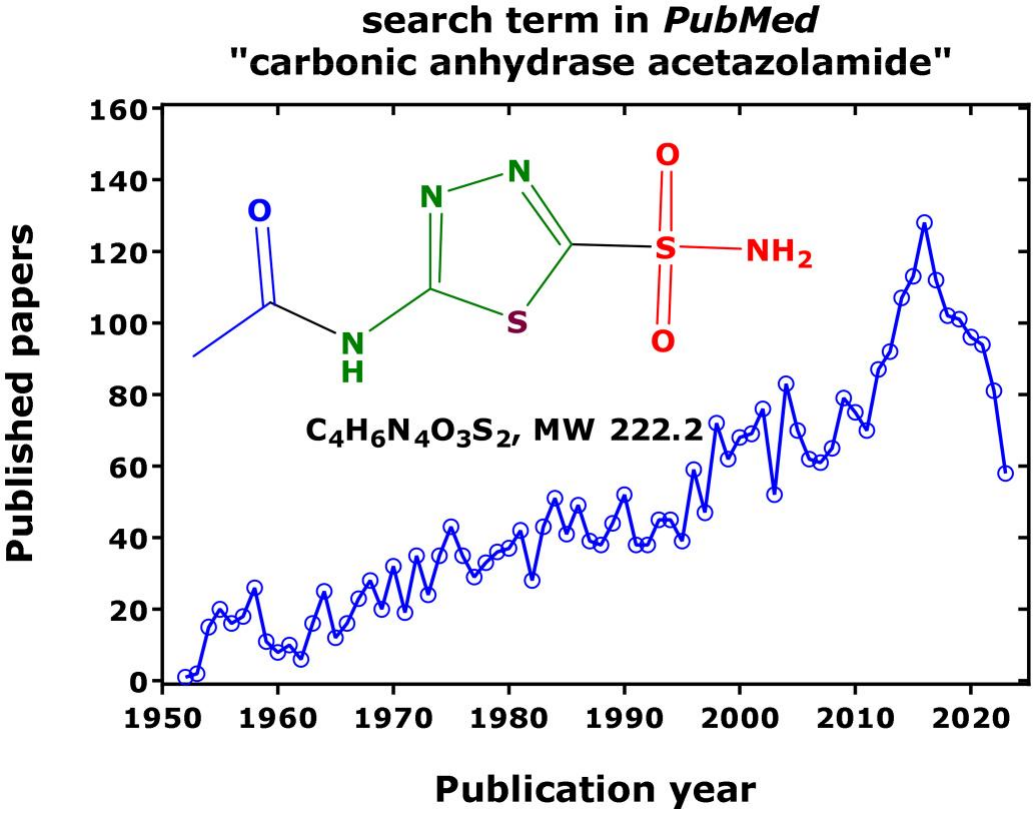
Published and archived articles in *PubMed* (https://pubmed.ncbi.nlm.nih.gov/) using the search term “carbonic anhydrase acetazolamide” 1952 to date (2023) and (date of search, 26 September 2023). As much as 3504 articles were published with a mean yearly publication rate of 49 articles. The number of articles using the term “carbonic anhydrase” amounts to 19,193. Inset shows the chemical structure of non-protonated AZM (*N*-(5-sulfamoyl-1,3,4-thiadiazol-2-yl)acetamide).

Acetazolamide (i.e. *N*-(5-sulfamoyl-1,3,4-thiadiazol-2-yl)acetamide, an organic sulphonamide R-SO_2_-NH_2_, p*K*_a_ 7.2) is one of the oldest therapeutically used drugs. Nevertheless, acetazolamide (AZM) and its action on carbonic anhydrases is still of steadily increasing, broad scientific interest (Figure 1). The number of research articles that appeared thus far in *PubMed* using the search term “carbonic anhydrase” amounts to 19,193 (4669 for CA II; 843 for CA IV; 2669 for the cancer-associated CA IX), and to 3504 using the term “carbonic anhydrase acetazolamide”, pointing out to a close relation between drug and target enzyme. The greatest hits were observed for the combination of AZM with CA II (535), followed by CA IX (187) and CA IV (155).

The biochemistry and pharmacology of the carbonic anhydrase family have been repeatedly reviewed by several authors. Human carbonic anhydrase (hCA) contains a coordinately bound Zn^2+^, which is absolutely required for enzymatic activity and its inhibition by sulphonamides such as AZM^3^^,^^4^. The mechanism of CA activity is assumed to start with the nucleophilic attack of the Zn^2+^-coordinated hydroxyl ion (^–^OH) that stems from an originally Zn^2+^-coordinated H_2_O molecule on the partially positively charged carbon atom of CO_2_ (^δ–^O = C ^δ+^= O^δ–^). Sulphonamides bind to the Zn^2+^ of carbonic anhydrase via their deprotonated -SO_2_NH_2_ functionality thus blocking the CA-catalysed reaction. With a *k_cat_*/*K*_M_ of 1.5 × 10^8^ M^−1^ s^−1^, CA II is one of the most active enzymes known, of which the activity is approaching the limit of diffusion-controlled reactions^5^. CA II has a turnover frequency of 10^6^ s^−1^, i.e. it is 10^7^ times faster than the uncatalyzed reaction. The *k_cat_* value for the CA-catalysed protonation of bicarbonate is about 4 × 10^5^ s^−1^.

AZM belongs to the class of diuretics, which are classified due to the primary sites of their pharmacological actions in the nephron^6^. AZM acts in the proximal convoluted tubule, where mainly two carbonic anhydrases are expressed. In addition to CA II and CA IV, there are other CA isozymes in the human kidney such as CA XII and CA XIV (see below). AZM is about six times more strong as an inhibitor of the cytosolic human (h) hCA II (*K*_i_, 12 nM) than of the membrane-bound hCA IV (*K*_i_, 74 nM)^5^. Approximately 85% of renal CA is considered to be CA II and 15% to be CA IV. Complete inhibition of renal CA activity was reported to be associated with about 30% reduction of the excretion of the filtered bicarbonate load^7^. These observations suggested that additional, CA-independent transport mechanisms are considerably involved in the reabsorption of bicarbonate in the kidney.

In humans, ingestion of AZM results in an abrupt manifold (up to about 20-fold) and temporary, a few hours lasting maximum bicarbonate concentration in the urine. The urinary pH value is also (abruptly) increased up to about 7.8, but it remains at this value for almost 24 h. As an example, ingestion of a regular 250-mg AZM tablet by a healthy volunteer resulted in an almost linear increase of AZM concentration in the urine within the first 2 h after intake; in the subsequent 3 h, the excretion rate of AZM in the urine was relatively constant^8^. This behaviour is in line with the pharmacokinetic profile of AZM as reported in pharmacokinetic studies on orally taken AZM in humans. Studies observed relatively long-lasting, concomitant and almost linear concentration-time curves for circulating and urinary AZM in humans after reaching its maximum concentrations in blood and urine^9^^,^^10^. AZM administration is also accompanied by increased excretion in the urine of water (diuresis) and electrolytes (saluresis) including sodium (Na^+^), potassium (K^+^) and ammonium (NH_4_^+^) cations as well as phosphate (H_2_PO_4_^–^) anions. In blood, these changes are much less pronounced.

Abrupt and strong increases in the concentration of bicarbonate in the urine of healthy subjects were observed upon ingestion of AZM tablets at therapeutical doses^11–13^. The size and shape of the bicarbonate peak depended upon the dose and the type of the AZM formulation. A dosage of 6.3 mg/kg AZM (one and a half Glaupax tablet) resulted in a sharp maximum of about 40 mmol carbonate/mmol creatinine 2 h after ingestion^11^. A similar dosage of 5 mg/kg (one 500-mg retard Diamox capsule) increased abruptly the bicarbonate concentration to reach an almost 2-h lasting plateau of about 22–25 mmol carbonate/mmol creatinine after 2 h^11^. In both cases, urinary pH increased and reached a value of about 7.8, which remained constant for about 24 h.

Ingestion of AZM at therapeutical doses also increased temporarily the excretion rate of endogenous nitrite and nitrate, as well of orally taken exgenous ^15^N-nitrite as a sodium salt dissolved in tap water (0.31 and 0.50 µmol/kg)^11–13^. The highest creatinine-corrected excretion rates of ^15^N-nitrite and ^15^N-nitrate were observed at the same time, i.e. about 1.0 and 1.5 h after ingestion of AZM^13^. These observations suggested that renal CA activity may be involved in the reabsorption of filtered unlabelled and ^15^N-labeled nitrite (O = N-O^–^, p*K*_a_ 3.4) and nitrate (O_2_N-O^–^, p*K*_a_ −1.37). The p*K*_a_ value of nitrous acid (O = N-OH) is comparable to that of carbonic acid^2^. The underlying mechanisms of the CA-dependent reabsorption of nitrite and nitrate^11–13^ are still elusive.

This observation may be of particular physiological and pharmacological importance. Nitrite and nitrate are the major metabolites of nitric oxide (NO), which is one of the strongest endogenous vasodilators and inhibitors of platelet aggregation. Nitrite and nitrate are considered abundant NO reservoir in human body^14^. It is worth mentioning that CA II exerts nitrous anhydrase activity in human platelets^15^. It is assumed that O = N–O^–^ is first protonated by hCA II to O = N–OH, which is then converted to its anhydride O = N–O–N = O (N_2_O_3_). In aqueous systems, nitrous anhydride is very labile, not isolable and decomposes to nitrite. N_2_O_3_ is a strong nitrosylating species, notably of thiols such as cysteine and glutathione. This newly discovered activity of carbonic anhydrase may contribute to the bioactivation of endogenous and exogenous inorganic nitrite and could be therefore of particular physiological and pharmacological importance.

In urine, blood and the nephron (see below), the concentration of pharmacological AZM is about three orders of magnitude higher than the *K*_i_ values of AZM towards hCA II and hCA IV over several hours post ingestion^8–10^. From a biochemical point of view, and because AZM does not undergo appreciable metabolism^15^^,^^16^ that would decrease its concentration in carbonic anhydrase-rich tissues such as the kidney, renal hCA II and hCA IV activity should be completely inhibited after ingestion of AZM at pharmaceutical doses. Yet, this is obviously not the case. This apparently paradoxical behaviour of AZM may suggest that the pharmacodynamic and pharmacokinetic properties of this drug, notably in the kidneys, are not yet fully explored. The high binding degree of AZM of about 93 to 96% to human proteins^16^^,^^17^ is likely to decrease the concentration of free AZM to a high extent, thus increasing the actual *K*_i_ value *in vivo* by several orders of magnitude to reach values that correspond to steady state plasma concentrations of AZM^17^. However, lower plasma protein binding degrees of the order of 49% for AZM has been reported in healthy subjects^10^. Also, AZM may influence directly or indirectly the activity of renal organic and inorganic ion transporters. In addition, the bicarbonate concentration at the apical membrane of the proximal tubule are in the lower mM-range. Thus, the hydration activity of hCA II and hCA IV may, at least in part, be inhibited by mM-concentrations of bicarbonate/carbonate: *K*_i_, 85 mM/73 mM for hCA II and *K*_i_, 6.6 mM/5.7 mM for hCA IV^18^. Protein and AZM concentrations are quite different at the luminal and basolateral membrane of the proximal tubule. It is therefore thinkable that the activity of the particular hCA IV is strongly inhibited by bicarbonate than the cytosolic hCA II activity in the proximal tubule.

At physiological concentrations, several L-amino acids and amines have been shown to activate many classes of CA, including hCA II and hCA IV^19^. This may be of particular significance because more than 95% of filtered amino acids are reabsorbed in the proximal tubule, i.e. the main site of expression and action of hCA II and hCA IV^20^. Many renal transporters are dependent on Na^+^ and H^+^, and AZM may, therefore, influence the urinary excretion of Na^+^ and H^+^ by those transporters^20^. The reabsorption of certain amino acids including arginine and its metabolites asymmetric dimethylarginine (ADMA) and symmetric (SDMA) dimethylarginine take place in the proximal tubule and in other segments of the nephron^21^. In addition, AZM has been reported to inhibit the activity of other renal enzymes such as glutamyltransferase^22^. All these effects together may result in the transient actions of AZM (acute administration) on the urinary excretion of electrolytes and even in the development of tolerance and resistance to AZM (chronic administration). Development of tolerance to drugs is a common and serious phenomenon in long-term therapy. The underlying mechanisms of these phenomena are still enigmatic for many classes of drugs such as nitroglycerine, on organic nitrate^23^. Two very early studies reported on tolerance/resistance development to AZM because of loss of diuretic action during continued administration of the drug^24^^,^^25^.

## Aims of the present work

The aim of the present work was to give a retrospect on AZM and carbonic anhydrase, and to review and discuss the literature on the pharmacology of AZM in humans reported in the *PubMed* (https://pubmed.ncbi.nlm.nih.gov/) over the past seven decades (1952 to 26th September 2023). The focus of the work is on the extraordinary pharmacokinetics and pharmacodynamics of AZM with respect to its main effects in the kidney, i.e. inhibition of renal hCA II and hCA IV activity, based on measurements of CO_2_, bicarbonate, pH, Na^+^, K^+^, Cl^–^ and other electrolytes in urine, serum, plasma and erythrocytes.

The effects of pharmacological AZM on the excretion of endogenous amino acids, inorganic nitrite, inorganic nitrate and malondialdehyde, which is a widely measured biomarker of oxidative stress, notably lipid peroxidation of polyunsaturated fatty acids including arachidonic acid^26^, have been little investigated so far. Amino acids are zwitterionic substances. At physiological pH values of blood and urine, nitrite and nitrate are permanently negatively charged species. Malondialdehyde (MDA, CHO-CH_2_-CHO) is an organic C–H acidic acid (p*K*_a_ 4.5). In the present work, we considered results on acute effects of pharmacological AZM ingested by a healthy young volunteer on the excretion of amino acids, nitrite, nitrate, malondialdehyde, and creatinine, in parallel with the measurement of AZM in urine^8^.

The present work also attempts to provide an explanation for the apparently paradoxical effects of acutely and chronically administered pharmacological AZM in the light of its strong inhibitory potency on its main target enzymes in human kidney, i.e. hCA II and hCA IV, and its remarkable pharmacokinetic profile including lacking metabolism/biotransformation.

## Acute effects of ingested acetazolamide on the urinary excretion of physiological substances in a healthy volunteer

### Pilot study and analysis

Urine samples had been collected in a previously reported study, in which a healthy volunteer (female, 29 years of age, 70 kg), ingested a 250-mg AZM tablet (Acemit^®^ from medphano, Germany)^8^. Immediately before (0 h) and at several times after drug administration, urine samples were collected (up to 5 h) in polypropylene bottles pre-cooled in an ice bath. The pH value of the urine samples was measured immediately by an pH electrode. Urine specimens were aliquoted, and the samples were analysed for AZM, nitrate, nitrite, creatinine, and malondialdehyde by gas chromatography-mass spectrometry (GC-MS) methods^27^. The concentration of amino acids was determined by GC-MS in 10-µL aliquots of urine^28^. Nitrate, nitrite, creatinine, MDA and AZM were analysed twice, i.e. without acidification and with acidification of the urine samples with 20 wt% acetic acid^8^^,^^29^. The urinary concentrations of AZM were measured by GC-MS in 50-µL aliquots as described previously^8^. The urinary excretion rates of the analytes were corrected for the urinary excretion rate of creatinine. They are expressed as µmol of analyte per mmol of creatine (µmol/mmol, i.e. µM/mM). As the GC-MS method used to determine the concentration of urinary amino acids cannot discriminate between the amino acids asparagine and aspartate (Asn, Asp), citrulline and ornithine (Cit, Orn), glutamine and glutamate (Gln, Glu), and leucine and isoleucine (Leu, Ile), the summed concentration of these amino acids is reported^28^. Statistical analyses were performed and graphs were prepared using GraphPad Prism 7 (San Diego, USA). Time series analysis using ARIMA (AutoRegressive Integrated Moving Average) algorithm was performed using SPSS.

## Effects of acetazolamide on nitrite, nitrate and creatinine excretion

**Figure 2.**
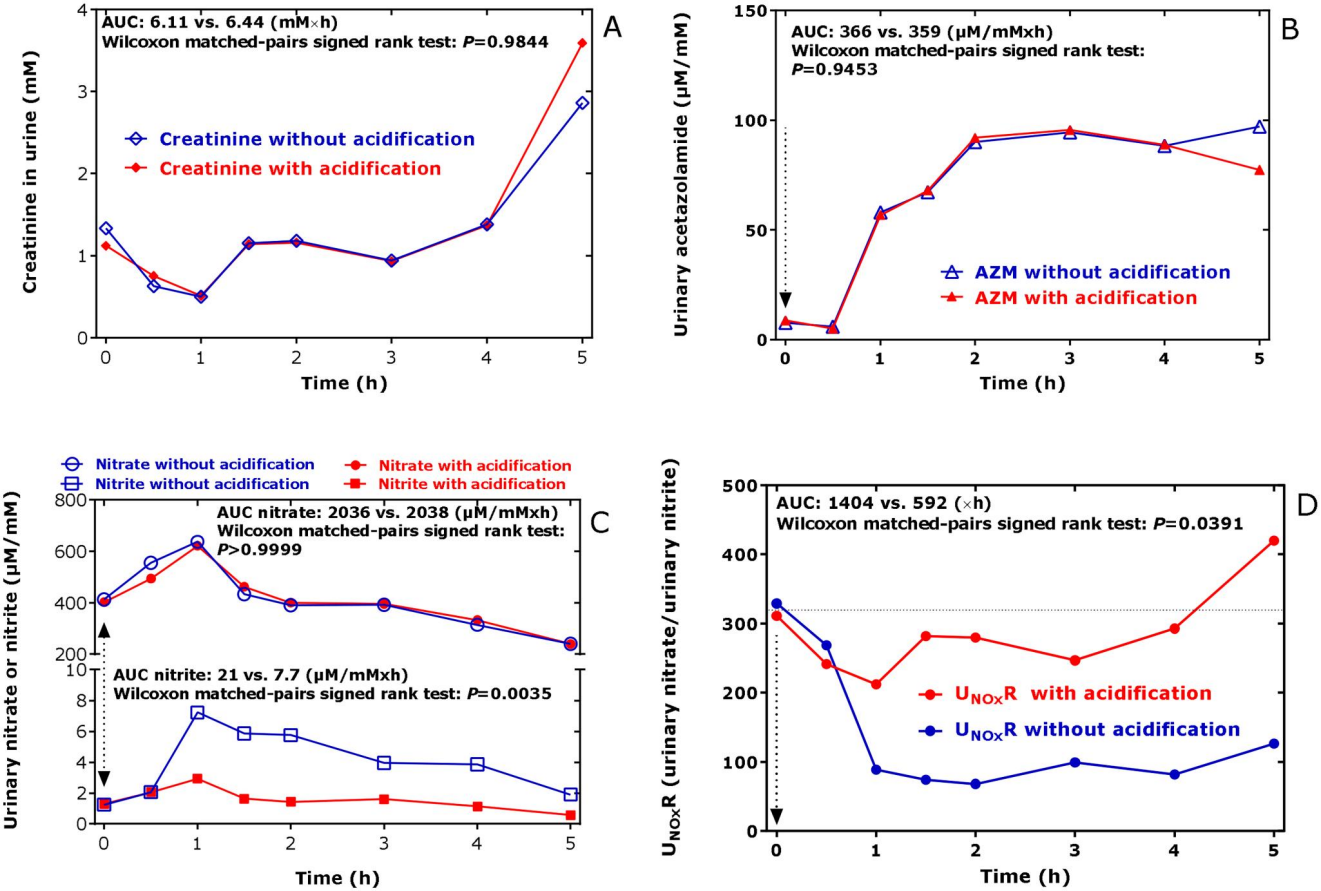
Time course of (A) the creatinine concentration in the urines samples collected in the study, of (B) the creatinine-corrected excretion rate of AZM (AZM), of (C) the creatinine-corrected excretion rates of nitrate and nitrite, and of (D) the urinary nitrate-to-urinary nitrite (U_NOx_R) in the respective urine samples without (blue) and with acidification (red) using 20 wt% acetic anhydride. Acidification of urine is required for the accurate measurement of nitrite^12^, but not for other analytes including nitrate, creatinine, AZM and amino acids. AUC, area under the curve. AZM, acetazolamide.

The urinary pH value was 7.1 at the start (0 h) and increased to the maximum value of 7.65 at 1.5 h, which remained almost unchanged until 5 h^8^. The creatinine concentration in the urine decreased to reach its minimum value of 0.5 mM at 1.5 h after AZM ingestion and increased thereafter to reach its baseline value (Figure 2(A)). There was no correlation between creatinine concentration and pH value of the urine samples (*r* = −0.098, *p* = 0.826). The areas under the curve (AUC) did not change upon acidification, indicating no effect of acidification on the GC-MS analysis of creatinine (Figure 2(A)). The temporary decrease in the concentration of creatinine in the urine samples is most likely due to elevated diuresis during the first hour upon AZM ingestion. Due to changes of the creatinine concentration in the urine over time, the urinary concentrations of all analytes were corrected by dividing the analyte concentrations with the respective creatinine concentration.

The highest concentration of AZM measured in the urine samples of the study was 278 µM. After a lag-time of 0.5 h, the creatinine-corrected excretion rate of AZM increased almost linearly (by 64 µmol/mmol/min) up to the value of 96 µmol/mmol at 2 h after ingestion; thereafter, the creatinine-corrected excretion rate of AZM remained almost unchanged at this level for the next 3 h (Figure 2(B))^8^. The AZM concentrations in the urine samples did not change upon acidification of the urine samples, indicating no pH-dependence of the GC-MS analysis of AZM in the urine samples (Figure 2(B)). The AZM concentrations in the urine samples measured are in agreement with previously reported data (see References below and citation in Ref.^8^), indicating a relatively constant and long-lasting excretion of unchanged AZM in the urine. We did not draw blood for the measurement of AZM in plasma samples. Other studies reported relatively constant circulating concentrations of AZM for many hours after AZM ingestion at therapeutical doses (see References below and citation in Ref.^8^).

The creatinine-corrected excretion rates of nitrate and nitrite changed over time. Maximum values were obtained from both anions each 1 h after AZM ingestion (Figure 2(C)). Acidification of the urine samples did not change the urinary nitrate excretion rates, while the nitrite excretion rates were considerably higher when the urine samples were not acidified post collection (Figure 2(C)). This observation suggests that nitrite may occur in human urine as an acid-labile bicarbonate adduct (e.g. O=N–O–C(=O)–O^–^) in the presence of higher bicarbonate concentrations^12^. The molar ratio of the nitrite values without acidification and those with acidification reached values that were 2.5–4 times higher. The molar ratio of nitrate-to-nitrite in urine, i.e. U_NOx_R, was suggested as a measure of nitrite-dependent CA activity in the kidney^30^. U_NOx_R decreased more strongly when the urine samples were not acidified (Figure 2(D)) due to the effect of urine acidification on nitrite concentration. The U_NOx_R values calculated for the acidified samples did not increase in the time window 1–5 h after AZM ingestion, whereas those measured in the acidified urine samples increased over time (Figure 2(D)).

The AUC values of the creatinine-corrected excretion rates of malondialdehyde (MDA) did not differ between acidified and non-acidified samples (2.6 and 3.2 µM/mM × h; *p* = 0.25; data not shown), indicating no effects of urine acidification on MDA analysis by the GC-MS method used^27^.

## Effects of acetazolamide on amino acids excretion

The creatinine-corrected excretion rates of nitrite, nitrate, MDA, amino acids and their metabolites analysed in the present study are summarised in Table 1. With the sole exception of MDA, the creatinine-corrected excretion rates of all other analytes increased temporarily upon AZM ingestion to reach maximum values after 0.5 h (21 analytes), 1.0 h (5 analytes), and 1.5 h (1 analyte) with some overlap for certain analytes.

**Table 1.** Urinary creatinine-corrected excretion rates (µmol analyte/mmol creatinine) of nitrite, nitrate and malondialdehyde (acidified urine samples), amino acids and some of their metabolites (non-acidified urine samples) before (0 h) and until 5 h of AZM intake (a 250-mg tablet Acemit^®^; Medphano, Germany) by a healthy female volunteer.

Time (h)	0	0.5	1	1.5	2	3	4	5
Nitrite	1.9	2.05	2.93	1.64	1.43	1.61	1.14	0.57
Nitrate	400	490	620	460	400	400	330	240
MDA	1.72	1.00	0.52	0.50	0.41	0.37	0.31	0.30
ADMA	2.7	3.84	3.3	3.11	3.68	3.19	2.73	3.77
SDMA	3.07	4.61	4.18	3.64	3.68	3.51	3.19	4.04
DMA	22.9	32.1	29.9	28.2	27.3	26.6	24.6	34.7
Ala	42.9	56.6	39.2	24.5	22.0	21.6	17.8	18.8
Thr	20.6	31.2	23.7	18.6	17.1	17.6	14.4	17.0
Gly	111	174	134	135	118	123	135	201
Val	6.48	11.5	9.82	9.41	9.16	7.55	6.85	8.33
Ser	40.8	64.4	67.0	37.5	37.1	41.1	32.2	34.4
Sarcosine	0.92	1.37	0.98	1.00	0.97	1.10	0.87	1.18
Leu + Ile	9.27	22.6	28.2	29.3	22.0	26.3	31.7	54.1
GAA	14.3	19.9	18.2	14.1	11.0	11.4	7.78	6.56
Asn + Asp	16.2	25.1	19.9	18.8	17.4	17.7	15.3	18.9
Hydroxy-Pro	0.51	0.85	0.85	1.22	0.55	0.77	0.63	0.63
Pro	2.09	3.33	2.89	1.81	2.62	2.42	2.06	2.45
Gln + Glu	99.5	130	106	91.8	85.5	77.4	71.7	112
Met	12.6	19.4	19.1	12.5	11.7	11.5	9.37	10.5
Orn + Cit	3.29	3.53	2.76	2.52	3.03	2.56	2.02	2.87
Phe	5.55	9.31	9.25	8.47	8.26	7.53	6.82	8.84
Tyr	11.6	17.3	15.5	14.7	14.4	13.8	12.2	15.4
Lys	582	809	668	397	335	293	193	191
Arg	2.15	3.29	2.69	2.35	3.00	2.08	1.68	2.21
hArg	0.15	0.22	0.22	0.20	0.17	0.13	0.10	0.12
Trp	8.78	12.1	10.2	9.09	9.41	9.91	8.04	9.83

Compared to the baseline values, the maximum excretion rates increased by factors of 1.1 (Met) to 3.2 (Leu + Ile) with a median [interquartile range] of 1.51 [1.40–1.58]. Spearman correlation between the creatinine-corrected excretion rate of nitrite and those of the other analytes revealed close correlations for the following analytes: GAA, 0.976; Lys, 0.952; Thr, 0.952; Met, 0.929; nitrate, 0.927; Ser, 0.905; MDA, 0.881; Ala, 0.881; and hArg, 0.850. These results indicate that the correlation is independent of the basicity/acidity of the amino acids.

The results of the time series analysis using ARIMA (AutoRegressive Integrated Moving Average) algorithm are listed in Table 2. AZM administration resulted in enhanced excretion of Leu + Ile (*r*^2^ = 0.640, *p* = 0.017), whereas the excretion rates of MDA (*r*^2^ = 0.687, *p* = 0.010), Ala, Thr, GAA, and Lys (*r*^2^ = 0.799, *p* = 0.003) decreased over time. The excretion rates of the remaining amino acids did not change over times or were marginal. The strongest effect was observed for Lys.

**Table 2.** Results of the time series analysis (ARIMA) with respect to the AZM-induced changes in creatinine-corrected urinary excretion rate of the analytes alongside their chemical structures and electrical charge at physiological pH values of plasma and urine.

Analyte	*r* ^2^	*p*	Structure	Net charge
Nitrite	0.335	0.13	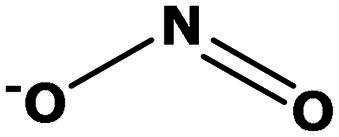	Negative
Nitrate	0.471	0.06	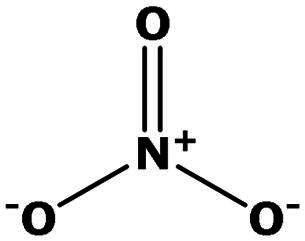	Negative
Malondialdehyde (MDA)	0.687	0.01	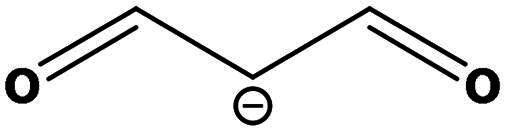	Negative
Asparagine (Asn)	0.137	0.37	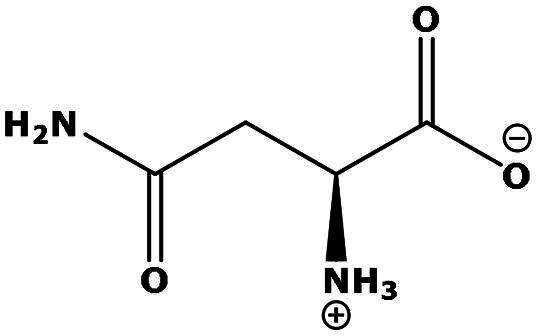	Neutral
Aspartate (Asp)			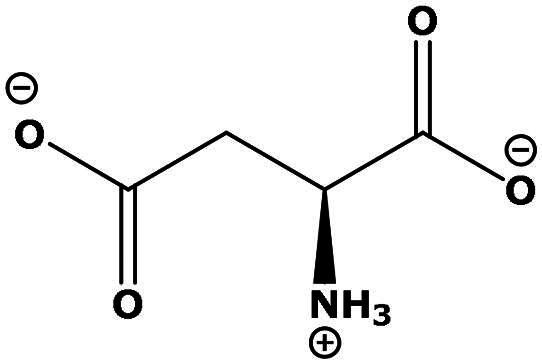	Negative
Glutamine (Gln)	0.202	0.26	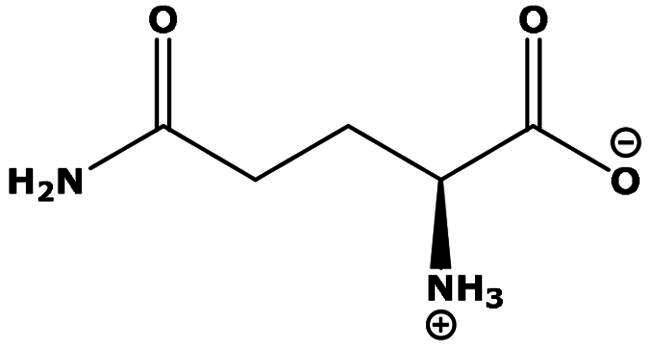	Neutral
Glutamate (Glu)			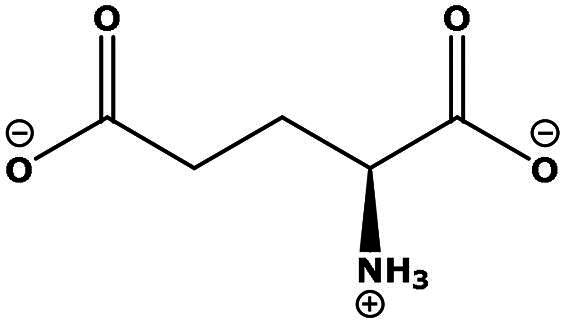	Negative
Alanine (Ala)	0.745	0.006	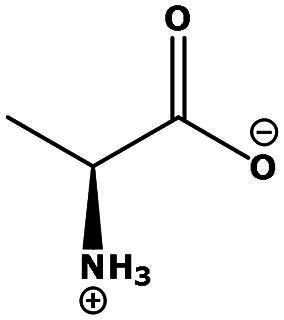	Neutral
Threonine (Thr)	0.505	0.048	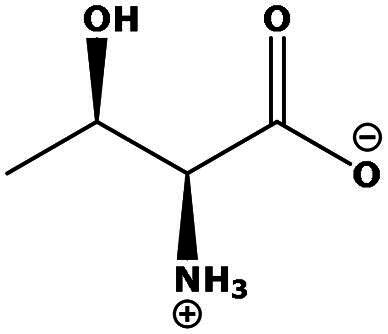	Neutral
Glycine (Gly)	0.1	0.37	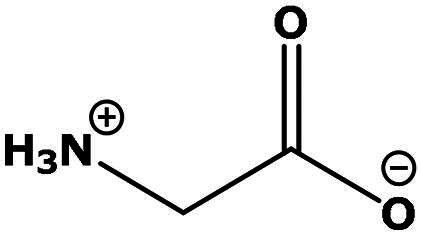	Neutral
Valine (Val)	0.09	0.46	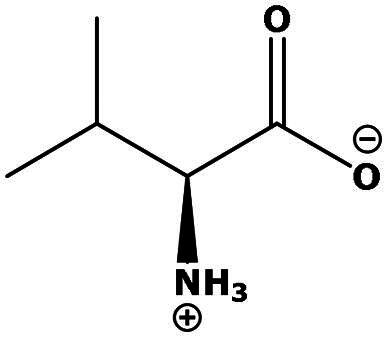	Neutral
Serine (Ser)	0.37	0.107	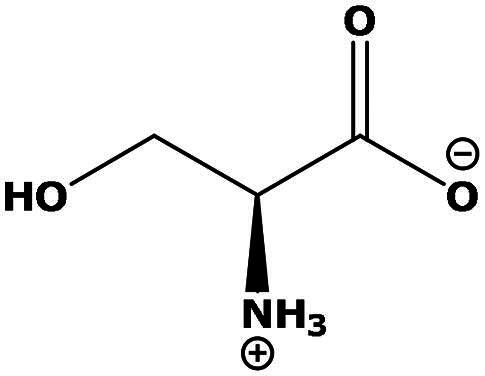	Neutral
Sarcosine (Sarc)	0.004	0.88	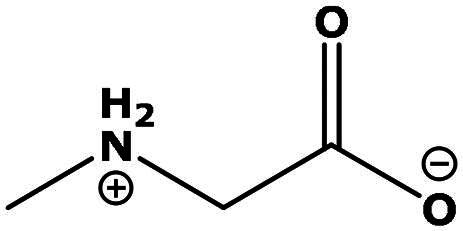	Neutral
Leucine (Leu)	0.64	0.017	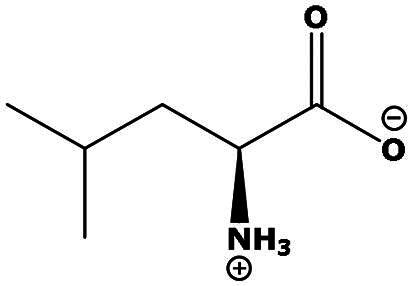	Neutral
Isoleucine (Ile)			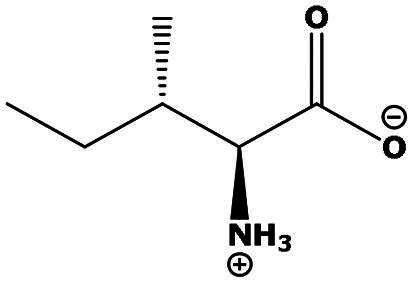	Neutral
Proline (Pro)	0.069	0.5	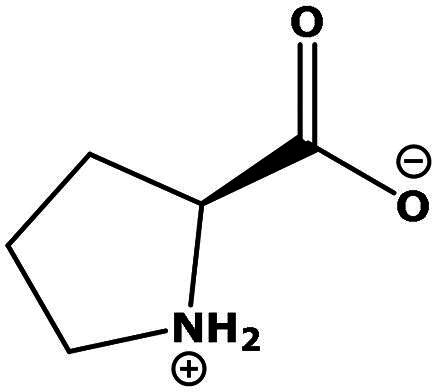	Neutral
Methionine (Met)	0.461	0.06	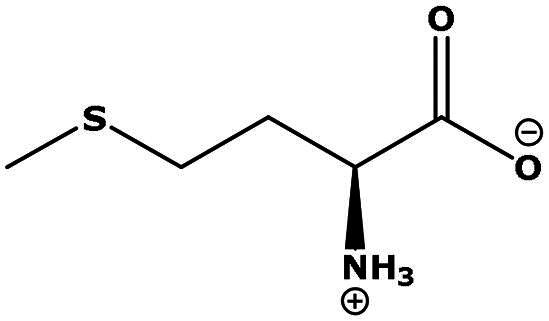	Neutral
Phenylalanine (Phe)	0.014	0.78	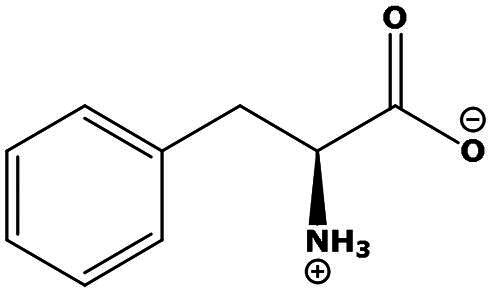	Neutral
Tyrosine (Tyr)	0.05	0.87	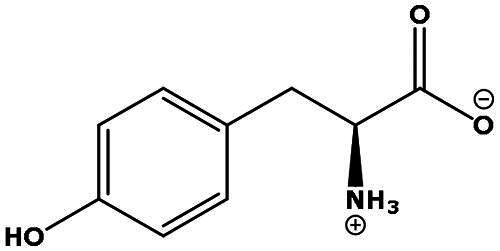	Neutral
Tryptophan (Trp)	0.107	0.424	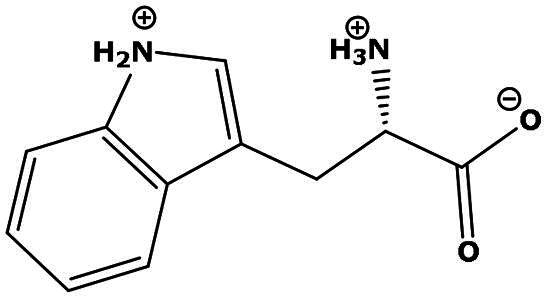	Neutral/positive
Ornithine (Orn)	0.424	0.08	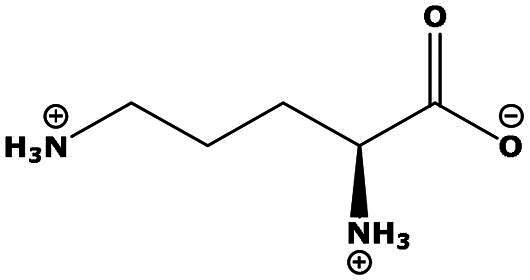	Positive
Citrulline (Cit)			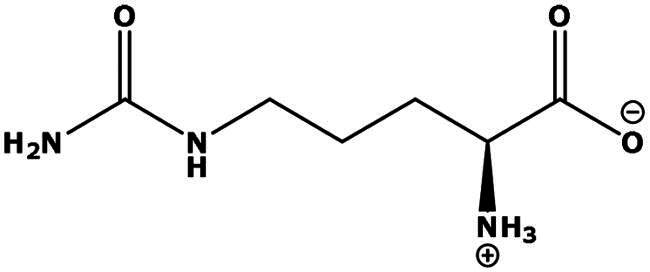	Neutral
Guanidinoacetate (GAA)	0.745	0.006	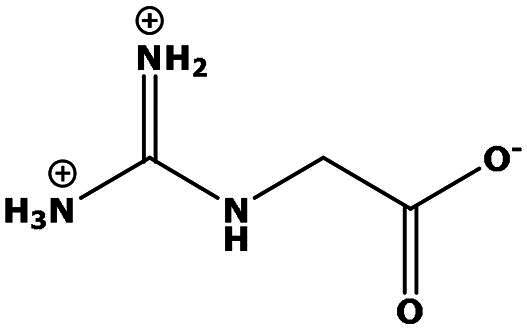	Positive
Lysine (Lys)	0.799	0.003	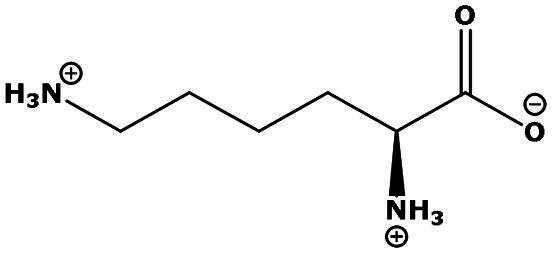	Positive
Arginine (Arg)	0.237	0.22	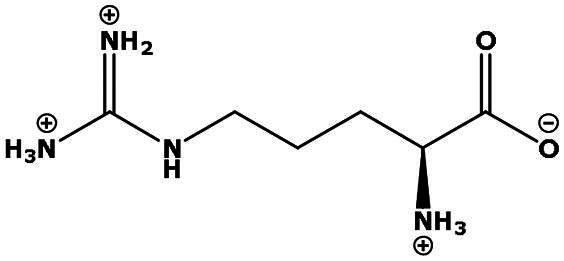	Positive
Homoarginine (hArg)	0.489	0.053	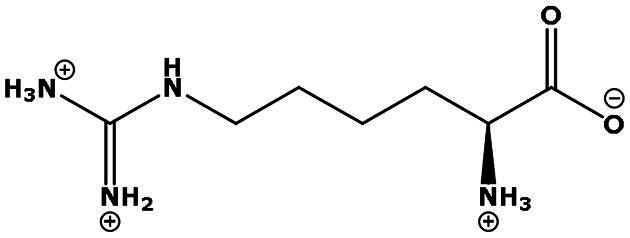	Positive
Symmetric dimethylarginine (SDMA)	0.017	0.7	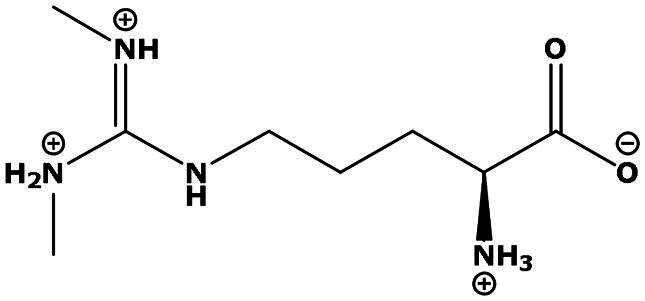	Positive
Asymmetric dimethylarginine (ADMA)	0.02	0.7	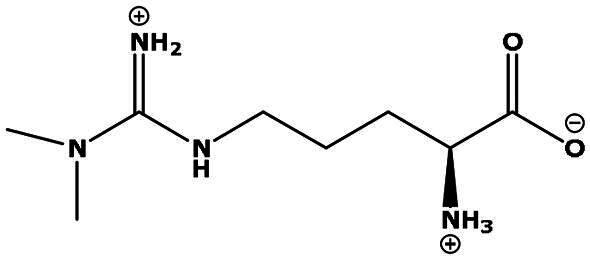	Positive
Dimethylamine (DMA)	0.067	0.5	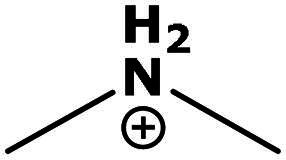	Positive
Creatinine	n.a.	n.a.	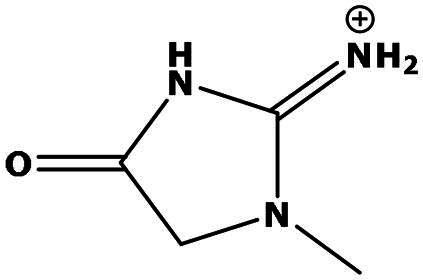	Positive
Acetazolamide (AZM)	n.a.	n.a.	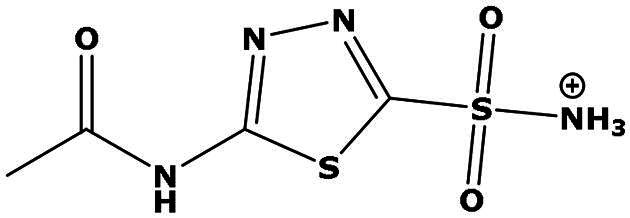	Positive

n.a.: not applicable.

## Metabolism of acetazolamide

Generally, it is assumed that ingested AZM is rapidly absorbed, is virtually not metabolised and is excreted unchanged in the urine. Recently two AZM metabolites were identified in hepatocyte incubations and three additional metabolites in urine and plasma samples of humans who ingested AZM^31^. Major transformations reported to include glutathione conjugation, glucuronidation, and *N*-acetylation. All these metabolites were detected in plasma 1.5 h after intake. Major metabolites were detected in urine from 0.25 to 24 h (last collection) after AZM intake^31^. The results of that study demand verification of the newly discovered metabolites of AZM and their quantitative relationship to unchanged AZM remains to be evaluated. Whether these metabolites possess pharmacological activity has not been reported. In the present work, it is considered that AZM does not undergo noticeable metabolism.

## Pharmacokinetics of acetazolamide

AZM in plasma, serum, red blood cells and urine has been measured by several methods including enzymatic assays, HPLC, GC-MS and LC-MS/MS. A selection of reported pharmacokinetic data of AZM is summarised in Table 3.

**Table 3.** Literature pharmacokinetic data of acetazolamide in humans.

Reference	Plasma (PL), serum (SE), erythrocytes (ER), blood (BL)				Urine			Administration and dose	Method	Remarks
1st Author et al.	*T*_max_	*C*_max_	*t*_½β_	*t*_½abs,_ *t*_lag_	*T*_max_	*N*_max_ *, C*_max_	*t*_½β_			
Ritschel et al.^9^^,^^10^	1–2 h	45 µM	4–6 h PL 9–12 h BL	0.7–1.0 h	n.r.	200 mg	9–12 h	a single 250 mg AZM tablet	HPLC	
Chapron et al.^16^	1.2 h (ER)	227 µM PL 173 µM ER	2.5 h PL	n.a.	n.a.	0.40 mg/min (at 1h) 0.05 mg/min (at 7h)	2 h	5 mg/kg i.v.	HPLC	Altitude non-linear
Yano et al.^17^	2 h	55 µM PL	7 h PL	1.1 h, 0.5 h	n.r.	n.r.	n.r.	125–500 mg b.i.d. oral	HPLC	IOP
Tsikas et al. (this work)	n.m.	n.m.	n.m.	n.m.	2 − 5 h	64 µM/mM/min		a single 250 mg AZM tablet	GC-MS	Healthy
Busardò et al.^31^	n.r.	n.r.	n.r.	n.r.	1 h	260 µM/mM	3 h	a single 250 mg AZM tablet	LC-MS/MS	Glaucoma
Hossie et al.^33^	1.0 h 2.5 h	6.3 µM 5.9 µM	6 h 6 h	n.r.	n.m.	n.m.	n.m.	1) single 250 mg AZM is solution 2) a 500-mg sustained-release tablet	HPLC	Healthy
Campíns-Falcó et al.^34^	n.m.	n.m.	n.m.	n.m	n.r.	35 µM, 19 µM, 2.5 µM at 8 h, 24 h, 48	9 h	a single 250 mg AZM tablet	HPLC	Healthy
Chapron et al.^32^	n.r.	227 µM 0 h 24 µM 5 h	n.r.	n.a.	n.r.	320 mg, 0.9 µmol/min	n.r	320 mg AZM i.v.	HPLC	Healthy
Hampson et al.^35^	1 h	4.5 µM	24 h	n.r.	n.r.	512–1024 µg/h	16 h	15 mg AZM oral	LC-MS/MS	Healthy
Yacatan et al.^37^	0.2 h 8 h	23 µM 82 µM	6 h 10 h	n.r.	n.m.	n.m.	n.m.	250 mg AZM different tablets	Enzymatic	Healthy
Straughn et al.^38^	0.5 h 3.0 h	4.5 µM 9.5 µM	3.8 h 9.8 h	n.r.	n.m.	n.m.	n.m	275 mg AZM different tablets	Enzymatic	Healthy

n.r., not reported; n.m., not measured

Intravenous injection of AZM (5 mg/kg) in elderly and young healthy subjects resulted in plasma AZM concentrations of 40–50 µg/mL (182–227 µM) and similar excretion rates (3–4 mg/min, 13.6–18.2 µmol/min) after 1 h. The concentration of AZM in the red blood cells was higher in the elderly compared to the young subjects (38 vs 24 mg/mL, 173 vs 109 µM)^16^. This study showed that the urinary excretion rate or AZM very closely parallels the concentration of AZM in plasma ultrafiltrate, indicating a dependence of AZM clearance on plasma protein binding.

Orally administered AZM (250 mg) reached maximal plasma concentrations of about 104 µM; its elimination half-life is about 4–6 h from plasma and about 9–12 h from blood as measured by HPLC^9^^,^^10^. The mean maximum concentration of AZM was determined to be 520 µg/mg creatinine corresponding to 260 µmol/mmol in urine about 1 h after AZM ingestion as measured by LC-MS/MS^31^.

Intravenously administered AZM (320 mg) resulted in an initial plasma AZM concentration of 227 µM, whereas the initial urinary excretion rate of AZM was determined to be 9.1 µmol/min as measured by HPLC^32^. After 5 h of administration, the plasma AZM concentration was about 24 µM in plasma, whereas the urinary excretion rate of AZM was determined to be 0.9 µmol/min^32^. In erythrocytes, higher concentrations of AZM were measured compared to plasma.

A maximum AZM plasma concentration of 16 µg/ml (7.3 µM) was measured at about 1 h after oral administration of 250 mg AZM in solution, whereas a maximum AZM concentration of 13 µg/ml (5.9 µM) was measured after about 2.5 h with a 500-mg sustained-release AZM tablet as measured by HPLC, with an estimated elimination half-life of about 6 h in both cases^33^.

After oral administration of a 250-mg AZM tablet, the mean urinary concentration of AZM was determined to be 78 µg/mL (35 µM) after 8 h, 41 µg/mL (19 µM) after 24 h and 5.6 µg/mL (2.5 µM) after 48 h of administration as measured by HPLC, corresponding to an elimination half-life of about 9 h from the urine^34^.

Oral administration of a microdose AZM of 15 mg resulted in a longer half-life elimination from plasma of about 24 h compared to therapeutic doses as measured by LC-MS/MS^35^. A maximum plasma AZM concentration of about 1.0 µg/mL (4.5 µM) was measured after 1 h of ingestion. Daily oral administration of 15 mg AZM resulted in elimination rates of 512–1024 µg/h in the urine. The half-life of the decline of the urinary excretion rate of AZM was determined to be 16 h after the last dose^35^. This and other studies suggested that AZM can permeate the red blood cell membrane and accumulate in the red blood cells, possibly explaining the pharmacokinetics of AZM in blood and urine even at microdoses.

In the rat, i.v. AZM infusion resulted in AZM concentrations in the Bowman’s space of 80 µM and 180 µM at infusion rates of 20 mg/kg and 50 mg/kg, respectively, as measured by HPLC^36^. The corresponding AZM concentrations in the end-proximal tubule were 240 µM and 470 µM^36^. At both doses, secretion accounted for most of the rise in the AZM concentration in the proximal tubule lumen. These findings indicate that AZM may reach considerable concentrations in the nephron including the proximal tubule, which is reached in hCA II and hCA IV.

Yakatan and colleagues performed bioavailability studies on healthy adult males using five different 250-mg AZM tablets^37^. AZM concentrations were measured enzymatically. Maximum plasma AZM concentrations were reached between 0.2 h and 8 h and ranged between 5 and 18 µg/mL (23–82 µM). The AUC values ranged between 45 and 168 µg/mL × h, indicating considerable bioinequivalence. With all tablets and 24 h after ingestion, mean plasma AZM concentrations of the order 1.5 µg/mL (7 µM). The elimination half-life is estimated to be 6–10 h^37^.

Straughn and colleagues performed similar studies on 12 young male volunteers using four different AZM tablets corresponding to a dose of 275 mg^38^. Plasma AZM concentrations were measured enzymatically. Maximum plasma AZM concentrations ranged between 0.5 h and 3 h and amounted to 10 to 21 µg/mL (4.5–9.5 µM). With all tablets and 25 h after ingestion, mean plasma AZM concentrations ranged between 0.8 and 1.3 µg/mL (3.6 and 5.9 µM). The elimination half-life of AZM is estimated to be 3.8–9.8 h. The AUC values ranged between 101 and 116 µg/mL × h indicating comparable bioequivalence^38^.

AZM is used extensively in equine veterinary medicine to control the effects of hyperkalemia. Alberts and colleagues investigated the pharmacokinetic of AZM after intravenous and oral administration in six horses^39^. Plasma AZM concentrations were determined by HPLC after extraction of AZM with ethyl acetate. Oral administration of AZM (8 mg/kg) resulted in rapid absorption. Mean maximum plasma AZM concentrations of 1.9 ± 1.1 µg/mL (about 9 ± 5 µM) were reached after 1.6 ± 1.2 h. The plasma concentration of AZM was about 0.2 µg/mL (0.9 µM) 24 h after administration. After i.v. injection of AZM (4 mg/kg), the first plasma AZM concentration was determined to be about 12 µg/mL (55 µM) and to decrease with a half-life of about 7.6 h. The oral bioavailability of AZM was calculated to be 25%, which is much lower than in humans. Steady-state volume of distribution and total body clearance were calculated to be 0.4 L/kg (0.2 L/kg in humans^10^) and 2.26 L/kg/h, respectively.

## Physiology of carbonic anhydrases and pharmacology of acetazolamide

Carbonic anhydrases play a major physiological role in acid–base and other solute transport along the nephron. Cytosolic CA II and membrane-bound CA IV provide the “push” and “pull” for bicarbonate transport, respectively. CA II and CA IV exhibit one of the highest hydrase catalytic rates of the order 10^6^/s. The physiological function of CA II in renal H^+^/HCO_3_^–^ transport is assumed to be best understood by examining CA II interactions with specific transporters^40^. CA II and CA IV are associated with bicarbonate transporters (e.g. AE1, kNBC1, NBC3, and SCL26A6), and proton antiporter, NHE1, in a membrane protein complex called a transport metabolon^40^. CA II is considered to account for more than 95% of the CA activity in the kidney, whereas CA IV is assumed to contribute about 5%^41^. The contributions of other more recently discovered renal CAs have not been carefully evaluated to date. In the proximal tubule, basolateral CA IV is a glycosylphosphatidylinositol-anchored protein^42^. In a renal proximal tubular cell culture model, CA II was found to bind to the epithelial Na^+^/H^+^ exchanger NHE3 and to increase its activity, while AZM significantly decreased NHE3 activity^42^. At least 65% of filtered Na^+^ is reabsorbed in the proximal tubule. H^+^ is considered to be generated by the cytosolic CA II.

Aquaporin-1 (AQP1) is the main water channel expressed on apical and basolateral membranes of epithelial cells of the proximal tubule and is considered to have the capacity to reabsorb 90% of the glomerular filtrate^43^^,^^44^. Despite a huge capacity for water transport, transport of other solutes including urea, glycerol, or even protons has not been detected^44^. AZM inhibits osmotic water permeability by interaction with AQP1^45^. In rats, AZM was found to exert diuretic affect by regulating AQP1. AQP1 translocation and degradation were found to mediate the water transportation mechanism of AZM^46^. AZM induced carbonic anhydrase activity recovery and AQP1 reduction^46^. The debate over whether aquaporins are permeable to CO_2_ continues, with accumulating both supportive and contradictory evidence^47^.

From a pharmacological point of view, the activity of hCA II (*K*_i_, 12 nM) and hCA IV (*K*_i_, 74 nM) is potently inhibited by AZM^5^. In the renal proximal tubule, inhibition of the activity of hCA II and hCA IV by AZM results in increased excretion of bicarbonate due to its inhibited reabsorption. AZM was found to modulate AQP-1 and this action is believed to explain the diuretic effect of AZM^46^.

The concentration of intact AZM upon ingestion of therapeutic doses reaches several µM-levels in blood and in tissue. Studies showed that the concentration of AZM in blood parallels that in urine over several hours^9^^,^^10^^,^^47^. This means that the carbonic anhydrase activity of hCA II and hCA II is likely to be completely inhibited in the proximal tubule, thus resulting in high long-lasting excretion rates of bicarbonate. Yet, in practice, this is not the case, suggesting that the underlying processes are much more complex and still incompletely understood. Because the plasma protein binding of AZM is high (93%, 96%)^16^^,^^17^, differences in tissue protein concentrations is expected to determine the unbound fraction of AZM and consequently its actual inhibitory potency. This may explain the demand of high doses of the drug to exert pharmacological effects in vivo that correspond to the *in vitro* high inhibitory potency of AZM towards hCA II and hCA IV^5^. Benzolamide and AZM can penetrate membranes of red blood cells^48^ and possibly those of renal tubular cells. AZM could be a less effective inhibitor of the cytosolic CA II compared to the easier accessible membrane-bound CA IV^49^. An alternative explanation could be effects of AZM on other transporters in the kidney, which are associated with hCA II and hCA IV.

In rabbits, AZM was shown to possess a non-linear pharmacokinetic^50^, exerting pharmacological responses such as urine flow rate, sodium excretion rate and ocular outflow pressure, but not duration of intraocular pressure, being depending on logarithmic AZM dose^51^. The concentration of AZM in urine is estimated to be increased by the kidney by a factor of about ten^52^. At an AZM concentration of 100 µM, carbonic anhydrase activity of hCA II and hCA IV is calculated to be completely inhibited, i.e. by 99.99%^18^^,^^52^.

A selection of reported pharmacodynamic data of AZM is summarised in Table 4. In patients without and with respiratory acidosis, ingestion of a single dose of AZM (2.2–6.5 mg/kg), resulted in abrupt changes of several electrolytes within 1–2 h^53^. Four hours after ingestion, the urinary pH ranged between 7.26–7.65 (compared to 5.56–6.79 without AZM). Urinary bicarbonate concentration ranged between 16–244 mM (compared to 0.06–15.0 mM). Urinary sodium concentration ranged between 105–450 mM (compared to 13–197 mM), while changes in urinary potassium concentrations were moderate (34–158 mM compared to 21–103 mM)^53^. Also, Galdston studied prolonged administration of AZM to patients every six hours and found a significant fall in the concentration of bicarbonate in plasma and in a decrease of plasma pH (acidosis)^53^.

**Table 4.** Literature pharmacodynamics data of acetazolamide in humans.

Reference	Urine			Plasma, serum, erythrocytes			Administration and dose	Remark
1st Author et al.	HCO_3_^−^	Na^+^	pH	HCO_3_^−^	Na^+^	pH		
Ritschel et al.^9^^,^^10^	n.r.	n.r.	4.5–7.8 4.2–6.9 3.1–6.7	n.r.	n.r.	n.r.	a single 250 mg AZM tablet	
Tsikas, Chobanyan^11^	40 mM/mM 25 mM/mM	n.r.	5.5–7.5 5.1–7.6	n.r.	n.r.	n.r.	6.3 mg/kg 5.0 mg/kg (retard capsule)	GC-MS
Chobanyan et al.^12^	n.r.	n.r	5.1–7.5 (1h)	n.r.	n.r.	n.r.	5.0 mg/kg	GC-MS
Leaf et al.^24^			5.5–7.5			7.38–7.28	250 mg 4x daily	Tolerance
Hanley abd Platts^25^	3–140 mM	n.r.		25–19 mM	n.r.	7.40–7.35	e.g. 250 mg 4xdaily 3–4 days	Self-limiting effect
Tsikas et al. (this work)	n.m.	n.m.	7.1–7.65	n.m.	n.m.	n.m.	a single 250 mg AZM tablet	Healthy
Galdston 1955^53^	16–244 mM 0.1–15 mM	105–450 mM 13–197 mM	7.26–7.65 5.56–6.79	19–35 mM 21–44 mM	n.m.	7.21–7.39 7.26–7.43	single AZM dose (2.2–6.5 mg/kg); long term; drug-free	Patients
Seldin et al.^66^	68–121–118 mmHg		5.94–6.82–7.0 (max.)	11.4–24.8 mM		7.23–7.56	single i.v. injection of 250 mg AZM	Healthy young men
Martens et al.^71^	AZM improves decongestive response over the entire range of HCO_3_^−^ levels; the treatment response is magnified in patients with baseline elevated HCO_3_^−^ levels by specifically counteracting diuretic resistance							519 patients with acute heart failure
Berthelsen et al.^74^	1) 1–357 µmol/min 2) 2–578 µmol/min	n.r.	1) 5.35–7.60 2) 6.33–7.66	n.r.	n.r.	2) 7.47–7.43	1) increasing cumulative i.v. doses of AZM (0–10 mg/kg) 2) single i.v. AZM dose (5 mg/kg)	Critically ill
Lichter et al.^84^	Tolerance rates (1) vs 2): 95 vs 100% after 1 week 63 vs 84% after 6 weeks 26 vs 58% beyond 6 weeks						1) 250 mg AZM tablets 4xdaily, 1 and 6 weeks 2) 500 mg AZM Sequels 2xdaily, 1 and 6 weeks	Glaucoma Tolerance

n.r.: not reported; n.m.: not measured

In the rat, i.v. AZM infusion (20 mg/kg and 50 mg/kg), decreased absolute proximal H_2_O and CO_2_ reabsorption by 50% and 80%, respectively, whereas AZM decreased whole kidney fractional total CO_2_ reabsorption by 25%^36^. The concentration of Na^+^ and K^+^ in the plasma did not change markedly, while the urinary excretion rates of Na^+^ and K^+^ in the urine increased by a factor of 52 and 3, respectively^36^.

AZM has been shown to bind to carbonic anhydrase^54^. Salicylate, which is an organic anion, has been reported to influence the binding of AZM to serum proteins, thus influencing the uptake of AZM by red blood cells^54^. Albumin has been described to regulate the renal clearance of AZM by binding this drug and reducing the unbound fraction of AZM^55^^,^^56^. These observations demonstrate that the renal plasma clearance of AZM is sensitive to changes in plasma protein binding.

## Effects of continuous acetazolamide administration on Na^+^, K^+^, Cl^–^, NH_4_^+^

Leaf and colleagues investigated the effects of continuous AZM (Diamox^®^) administration to humans on electrolyte and acid-base balance in serum and urine^24^. Oral administration of 250 mg AZM four times daily over several days to patients and healthy humans resulted in small increases in Na^+^, K^+^ and Cl^–^, and in falls of pH in serum compared to the days before AZM ingestion (Table 4). In urine, pH and the concentration of Na^+^, K^+^ and Cl^–^ increased immediately, while the concentration of NH_4_^+^ decreased immediately. The highest Na^+^, K^+^ and Cl^–^ concentrations and the higher values of urinary pH were measured on day 1 and day 2 after administration. Urinary NH_4_^+^ concentration was lowest on day one after administration. After reaching the maximal and minimal values in the urine, Na^+^, K^+^ and Cl^–^ concentrations and the pH value decreased continuously, while urinary NH_4_^+^ concentrations increased steadily. Promptly on cessation of AZM administration, urinary Cl^–^ concentration increased and urinary NH_4_^+^ concentrations increased even above the values before drug administration, while urinary pH and the urinary concentrations of Na^+^ and K^+^ decreased continuously to reach the values before AZM intake. This early study reported on resistance to the diuretic action of AZM. Prompt development of resistance to AZM was observed: Four days of continuous AZM ingestion had no greater total effect on electrolytes than the first single dose. Intervals of one and two days between single doses failed to restore the initial response level^24^.

Hanley and Platts investigated the mode and rate of recovery from AZM action in healthy humans and in patients with heart failure (Table 4)^25^. This early study reported on resistance to the diuretic action of AZM. AZM (Diamox^®^, 250 mg, 400 mg or 500 mg) was administered orally as a single daily dose (at 0, 24 h, 48 h, 72 h, 96 h). On day one, urinary bicarbonate excretion increased 14- to 46-fold, whereas urinary NH_4_^+^ excretion decreased (1.7- to 4.6-fold). On the next days, the urinary bicarbonate excretion was much less pronounced, whereas urinary NH_4_^+^ excretion was higher than on the first day despite daily administration of AZM. On the subsequent recovery drug-free days, urinary bicarbonate was not measurable, while the urinary NH_4_^+^ concentration increased to values above the initial values^25^. In eight patients with congestive heart failure ingesting AZM (200 or 250 mg 4-h), the cumulative bicarbonate excretion increased to reach values ranging between 150 and 250 mM after about two days. Hanley and Platts concluded that 5–6 days will be required for full recovery from a series of AZM doses, and 2–4 days for recovery from an initial single dose. With respect to the self-limiting effects of AZM, one mode of action of AZM was proposed a kind of metabolic acidosis.

## Effects of acetazolamide on the excretion of MDA, amino acids, nitrite, and nitrate

Malondialdehyde (MDA) is a product of lipid peroxidation of polyunsaturated fatty acids including arachidonic acid^26^. In the present study, malondialdehyde (MDA) was the only analyte of which urinary excretion consistently decreased upon AZM ingestion (Table 1). Whether the sustainable decrease in urinary MDA excretion is due to the inhibition of MDA synthesis by AZM or due to inhibition of the renal MDA excretion by AZM is unknown.

MDA and thromboxane A_2_ (TxA_2_) are produced concomitantly mainly in the platelets^26^. MDA and TxA_2_ synthesis is decreased by inhibitors of cyclooxygenase (COX), notably by acetylsalicylic acid (aspirin)^26^. Some sulphonamides that inhibit CA activity have been reported to inhibit COX activity as well, most likely through their sulphonamide group^57^. In humans, cerebrovascular reactivity of infused AZM was found to correlate inversely with the plasma concentrations of the TxA_2_ metabolites TxB_2_ and 11-dehydro-TxB_2_, suggesting inhibition of TxA_2_ synthesis in the platelets^58^. Although MDA had not been measured in that study, it is possible that plasma MDA concentrations also correlated inversely with the AZM reactivity in the cerebellum.

The effects of AZM on the urinary excretion of amino acids in humans is insufficiently investigated and the available reports are inclusive. The reabsorption of amino acids from the urine occurs along the nephron^20^^,^^21^^,^^59^. Many of the renal transporters are dependent on Na^+^, H^+^ or Cl^–^. About 95–99% of all amino acids are reabsorbed in the proximal convoluted tubule and proximal straight tubule. Neutral amino acids represent 80% of the free plasma amino acids and are all transported by the luminal B0 AT1 transporter (SLC6A19). The basic system transports cationic amino acids together with cysteine. Glutamate and aspartate are transported by the acidic system, while the iminoglycine system transports proline, hydroxyproline, and glycine.

In an early study, Madsen and colleagues found that the concentration of amino acids and uric acid in the urine was maximum after 2 h of AZM ingestion (7–12 mg/kg), minimum after 6 h, and to increase slowly thereafter^60^.

Intravenous injection of AZM (5 mg/kg) in a metabolically healthy infant (female, 3.3 years old) increased the excretion rate of many amino acids in urine samples that were collected after 6 and 12 h (by 38% for the summed amino acids)^61^. The concentration of the amino acids in plasma remained almost unchanged^61^. In contrast, intravenous injection of AZM (5 mg/kg) in an infant with cystinuria (female, 1.5 years old) decreased the excretion of many amino acids (by 42% for the summed amino acids)^61^. The highest decrease in urinary excretion rate was observed for lysine. These effects disappeared when AZM was repeatedly administered.

Gougoux and colleagues performed detailed studies with AZM in normal and acidotic dogs^62^. The excretion of glutamine, glutamate and aspartate in the urine samples collected for four 10-min periods remained minimal before infusion (-40 to 0 min) compared to after infusion (0–40, 40–80, 80–120 min) of AZM (priming dose, 20 mg/kg; maintenance dose 20 mg/kg/h)^62^. In the normal dogs, urine pH increased from 6.90 to 7.65, 7.62 and 7.54, respectively. In kidney cortex, AZM (range, 0.1 mM–2 mM) inhibited the activity of alphaketoglutarate dehydrogenase and succinyl-CoA synthetase^62^. In the acidotic dogs, urine pH increased from 5.56 to 7.10, 6.99 and 6.91, respectively. These studies indicated that AZM may exert distinctly different effects on the reabsorption of amino acids in health and disease, especially in those being associated with impaired amino acid metabolism such as cystinuria and acidosis, and may exert inhibitory effects not only towards various enzymes in addition to carbonic anhydrase.

Investigations on the effects of AZM on nitrite and nitrate are very rare. In rats, i.v. bolus injection of AZM (50 mg/kg) increased the excretion rate of urine almost by a factor of 13, while the urinary excretion rate of Na^+^ was increased by a factor of 19 and that of K^+^ by a factor 3; AZM increased the fractional excretion of Na^+^ by a factor of 21^63^. That study showed that AZM increases acutely the excretion of nitrate + nitrite, i.e. the sum of nitrite and nitrate, by a factor of about 3. Less pronounced effects were observed with the diuretic furosemide (Na^+^, 15-fold; K^+^, 2.7-fold; fractional extraction of Na^+^, 16-fold; nitrate + nitrite, 1.7-fold)^62^. In that study, the excretion of amino acids had not been investigated.

In humans, renal CA II and CA IV are involved in the reabsorption of endogenous and exogenous nitrate and nitrite^11–13^^,^^63^. Sialin (SLC17A5) is a H^+^/nitrate cotransporter and a H^+^/sialic acid cotransporter in the plasma membrane^64^. In humans, sialin is encoded by the *SLC17A5* gene. This protein is widely expressed in various organs including kidney^64^^,^^65^. The transport of nitrate is also known to be mediated by chloride channels (CLC)^65^. As mentioned above, the excretion of Cl^–^ in the urine is increased by AZM, which is accompanied by a decrease in circulating Cl^–^ concentration^24^. It is unknown whether the transport of nitrate and nitrite by sialin is inhibited by AZM.

## Effects of acetazolamide on renal bicarbonate reabsorption

It is known for more than 70 years that the reabsorption of filtered bicarbonate by the kidney results from the secretion of cellular H^+^ in exchange for tubular Na^+^. At that time, the function of carbonic anhydrase was thought to maintain an adequate supply of H^+^ by accelerating the hydration of cellular CO_2_^66^. According to the widely accepted theory prevailing at that time, the secreted H^+^ was assumed to react with HCO_3_^–^ to form H_2_CO_3_, which then decomposes spontaneously to CO_2_ and H_2_O, thus reducing the concentration of HCO_3_^–^ in the filtered urine. Inhibition of carbonic anhydrase activity by AZM results in the formation of less protons, thus leading to higher concentrations of HCO_3_^–^ in the urine and to lower concentrations of H^+^ in the urine that results in alkalisation of the urine^66^. The inhibition of carbonic anhydrase activity to abolish HCO_3_^–^ reabsorption is known for long time to be incomplete. It has been hypothesised that the formation of H^+^ from the chemical, i.e. non-CA catalysed reaction, is an important source of H^+^ for the Na^+^/H^+^ exchange transport.

In healthy young men, intravenous injection of 250 mg AZM (Diamox^®^) resulted in an increase of the urine pH from 5.94 (period, 0–20 min) to 6.82 (period, 120–140 min) with a maximum pH value of 7.0 being measured in the period 60–80 min. The corresponding urinary PaCO_2_ values were 68, 121 and 118 mmHg. There was a linear relationship between reabsorbed HCO_3_^–^ and PaCO_2_ values despite marked inhibition of CA activity, suggesting that the non-CA-catalysed formation of H^+^ is a considerable contribution to reabsorption of HCO_3_^–0.66^.

Recently, a new theory of the role of CA IV in the kidney has been proposed and reviewed^67^. It was hypothesised that the role of renal CA IV is the minimisation of pH changes in nanodomains near the basolateral membrane^67^. This theory is based on the assumption that two of the NCBTs carry CO_3_^2−^ rather than HCO_3_^−^. In the lumina of the proximal tubule, the thick ascending limb and the distal nephron, NHE3 and vacuolar-type H^+^ pump (V-ATPase) secrete H^+^ that reacts with filtered HCO_3_^–^ to generate CO_2_ and H_2_O by the catalytic action of the apical CA IV. CO_2_ and H_2_O are transferred by AQP1 into the cytosol of the proximal tubule and converted to HCO_3_^−^ and H^+^ by the catalytic action of CAII^67^. Yet, the extent of contribution of AQP1 to the CO_2_ transport in the proximal tubule is unknown. Direct extracellular interaction between CA IV and the human NBC1 sodium/bicarbonate co-transporter has been reported^68^. Interactions of CA II with the Na^+^/H^+^ exchanger^69^ and CA IV with human AE1 Cl^–^/HCO_3_^–^ exchanger have also been reported^70^.

The effects of AZM (i.v. bolus injection, 500 mg/d for two days) on decongestion were investigated in 519 elderly patients with acute heart failure and volume overload in combination with oral maintenance therapy with at least 40 mg furosemide or an equivalent dose for at least one month^71^. Bicarbonate was determined in venous blood. Placebo resulted in increases of venous blood bicarbonate concentration, while AZM reduced the venous blood bicarbonate concentration. AZM decreased the mean congestive score more strongly in the patients who had higher baseline bicarbonate levels (>27 mM vs <27 mM). Combined use of proximal tubule and loop diuretics improved decongestive effectiveness, diuretic response, and shortened the length of stay. The authors interpreted these observations by assuming specific counteraction of poor loop diuretic response by AZM in the patients sub-group^71^. These findings seem to collaborate with the observation by Galdston on significant falls in the plasma concentration of bicarbonate upon long-term administration of AZM^53^.

The effects of high-molecular-mass carbonic anhydrase inhibitors on renal bicarbonate reabsorption have been investigated. In vitro investigations (isolated perfused rat kidney) with dextran-bound high-molecular-mass (about 67 kDa) cell-impermeable inhibitors of carbonic anhydrase revealed that CA IV is critical for normal reabsorption of bicarbonate^72^.

F3500 is a high-molecular-mass (3500 Da) carbonic anhydrase inhibitor, a polyoxyethylene derivative of the low-molecular-mass CA inhibitor aminobenzolamide^73^. The *K*_i_ values of F3500 are 140 nM towards CA II and 4000 nM towards CA IV. F3500 does not penetrate red blood cells. In rats, 30–60 min upon i.v. injection of 100 mg/kg of F3500 resulted in maximum HCO_3_^–^ concentrations of 40 mM after. In comparison, 30–60 min upon i.v. injection of the membrane-permeable carbonic anhydrase inhibitor aminobenzolamide (a CA II and CA IV inhibitor) resulted in maximum urinary HCO_3_^–^ concentrations of 105 mM^72^.

These observations suggest that under physiological conditions CA II and CA IV are necessary for maximum renal reabsorption of HCO_3_^–^. Luminal CA IV converts filtered HCO_3_^–^ to H_2_CO_3_ by using H^+^ provided by the cytosolic CA II.

## Immediate effects of acetazolamide in critically ill patients

Critically ill patients require diuretics to prevent or treat over-hydration. Berthelsen and colleagues investigated renal and respiratory effects of AZM in the critically ill^74^.

Six patients each had five i.v. bolus injections of AZM, with 20-min intervals, so that the cumulative dose after 20 min was 1 mg/kg, after 40 min 2.5 mg/kg, after 60 min 5 mg/kg, after 80 min 7.5 mg/kg and after 100 min 10 mg/kg^74^. Specimens of urine were collected in the 10 min preceding each of the injections, and 10–20 min after the last injection. Blood-gas tensions and acid-base balance were determined before each injection.

Urine volume excretion rate increased from 0.9 ml/min to 2.4, 3.6, 3.1, 2.9, 4.3 ml/min, respectively. Urine pH values increased from 5.35 to 7.45, 7.50, 7.56, 7.60, 7.60 ml/min, respectively. Urine bicarbonate excretion rate increased from 1 µmol/min to 163, 323, 346, 350, 357 µmol/min, respectively, indicating no further increases at the last three injections (Table 4)^74^.

The same researcher group also investigated the effects of intravenous administration of AZM to additional six patients (5 mg/kg)^74^. AZM resulted in mean increases of excretion rate of the urine volume from 0.8 ml/min prior to injection to 4.2 ml/min after 20 min of injection (5-fold). Urine pH increased from 6.33 to 7.66, while urine bicarbonate excretion rate increased from 2 µmol/min to 578 µmol/min (290-fold). Mean venous blood pH and serum K^+^ fall from 7.47 to 7.43, and 3.3 to 2.8 mM, respectively (Table 4)^74^. The concomitant transient inhibition of pulmonary CO_2_ excretion was small (4%) and considered of no clinical importance.

## Side effects of acetazolamide therapy

Schmickl and colleagues included 42 studies in their meta-analyses of side effects of AZM therapy (542 mg total daily dose, 6.9 mg/kg) in acute/chronic mountain sickness, sleep disorder breathing, and ophthalmology for 3 to 7 days^75^. The most common side effects of AZM were paraesthesias. Severe side effects were rare and largely avoided by careful patient selection (e.g. hypo-kalaemia occurs almost exclusively in patients co-treated with thiazide diuretics or angiotensin receptor blockers)^75^. AZM increased in part dose-dependently the risk of paraesthesia (39 studies), taste disturbance (23 studies), polyuria (22 studies), fatigue (14 studies), nausea (12 studies) and dizziness (10 studies)^75^.

Decreased libido^76^ and sexual impotence^77^ have been reported in subjects treated with AZM for glaucoma. Decreased libido completely reversed or markedly improved after discontinuation of the drug in all cases^76^. Gastric mucus and bicarbonate secretion are involved in mucosal protection^78^. In rats, AZM produced gastric ulceration^79^. Although the starting hypothesis for treating peptic ulcers with sulphonamide carbonic anhydrase inhibitors was wrong, the treatment turned out efficacious and this is due to inhibition of β-CA produced by *Helicobacter pylori*. β-CA are involved in crucial physiologic processes in the *Helicobacter pylori* bacterium^80^. Finding carbonic anhydrase inhibitors selective for the bacterial (β-CA) over the human (α-CA) enzymes may lead to efficient treatment approaches for peptic ulcers^81^^,^^82^.

## Tolerance and drug resistance to acetazolamide therapy

The development of tolerance to drugs is a common and serious phenomenon in long-term pharmacotherapy. The underlying mechanisms are still enigmatic for many classes of drugs such as the organic nitrates^23^.

Two very early studies reported on tolerance/resistance development to AZM (Table 4)^24^^,^^25^. Leaf and colleagues reported on prompt development of resistance to AZM upon continuous AZM ingestion. Four days of continuous AZM ingestion had no greater total effect on electrolytes than the first single dose. Intervals of one and two days between single doses failed to restore the initial response level^24^. Hanley and Platts investigated the mode and rate of recovery from AZM action in healthy humans and in patients with heart failure^24^. Hanley and Platts concluded that 5 to 6 days will be required for full recovery from a series of AZM doses, and 2 to 4 days for recovery from an initial single dose. With respect to the self-limiting effects of AZM, one mode of action of AZM was proposed a kind of metabolic acidosis^25^.

Also, Galdston studied prolonged administration of AZM to patients every six hours and found a significant fall in the concentration of bicarbonate in plasma and in decrease of plasma pH (acidosis)^53^.

In another early study, long-term administration of AZM to patients with glaucoma resulted in inconclusive results^83^. In a later study on patients with glaucoma, AZM given as 250 mg tablets every six hours or as 500 mg Sequels every 12 h resulted in different tolerance rates: 95 vs 100% at 1 week, 63 vs 84 at 6 weeks and 26% vs. 58% beyond 6 weeks (Table 4)^84^. Effects of AZM treatment on urinary excretion of electrolytes have not been reported in the above-mentioned two studies.

AZM has been used for decades in the treatment of epilepsy^85^^,^^86^. The exact mechanism of its epileptic action is not precisely known. Direct inhibition of carbonic anhydrase in neurons and attenuation of action potential firing were discussed. Despite its potential anti-seizure effects, AZM is rarely used due to perceived adverse events and the development of tolerance. Shukralla an colleagues concluded that the evidence from several observational studies may overestimate the efficacy of AZM in epilepsy^86^. In mice, the tolerance mechanism in epilepsy is considered to be due to an increased production of carbonic anhydrase and/or changes in phosphorylation of the enzyme and alterations in synaptic transmission with long-term use^86^. AZM appears safe and efficacious for seizure management despite tolerance development in long-term use^86^.

AZM, nitroglycerine and acetylsalicylic acid (aspirin) belong to the group of the oldest pharmacologically used drugs that are known to develop tolerance^23^^,^^75^^,^^86^^,^^88^. Tolerance development to AZM in its long-term use has been reported to include inhibition of AQP1^45–47^^,^^87^^,^^89^. Other carbonic anhydrase inhibitors such as dorzolamide are pharmacological less efficacious than AZM towards some isoforms (*K*_i_ 9 nM for hCA II; *K*_i_ 8500 nM for hCA IV for dorzolamide)^5^, but better tolerable than AZM as adjunctive therapy to the β-blocker timolol in patients with elevated intraocular pressure^89^.

## Summary and discussion

CO_2_ reacts slowly with H_2_O to HCO_3_^–^ and H^+^, and H_2_CO_3_ dehydrates much more rapidly to CO_2_ and H_2_O by a factor of about 600 (i.e. 23 s^−1^/0.039 s^−1^). Carbonic anhydrases catalyse the hydration of CO_2_ and produce HCO_3_^–^ and H^+^, most likely via intermediate formation of the extremely labil aand short-lived H_2_CO_3_. Carbonic anhydrases also catalyse the protonation of HCO_3_^–^ to generate CO_2_ and H_2_O, most likely via intermediate formation of H_2_CO_3_ as well.

**Figure 3.**
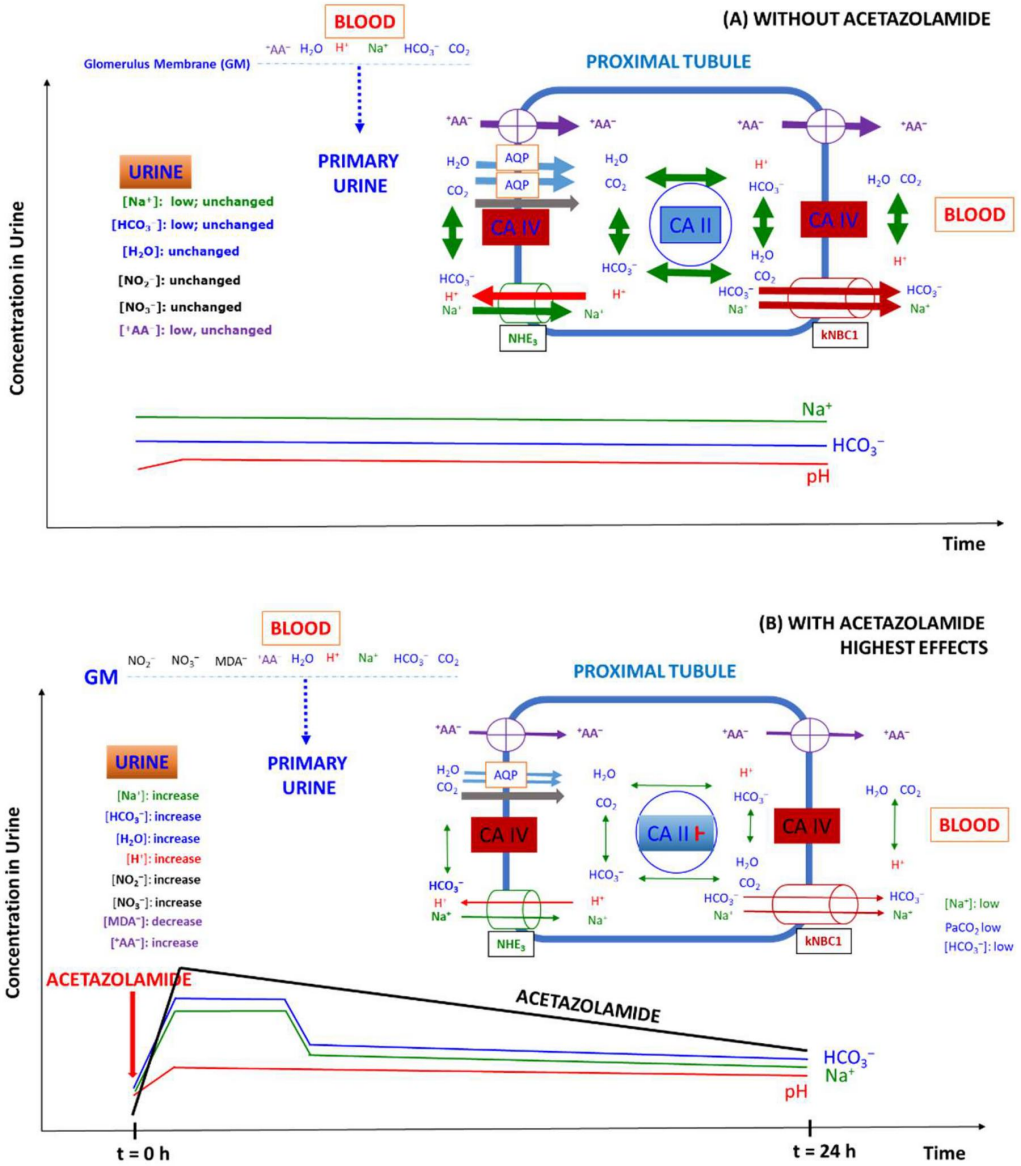
Simplified schematic of the role of carbonic anhydrase isoforms CA II (cytosolic) and CA IV (membrane-bound) and some transporters in the proximal tubule of the healthy kidney on the reabsorption of Na^+^, HCO_3_^–^, water, nitrite, nitrate, malondialdehyde (MDA) and zwitterionic amino acids (^+^AA^-^) under two different conditions: (A) Under physiological conditions, i.e. in the absence of AZM; (B) in the presence of AZM; AZM was taken orally at the time point zero as indicated by the vertical red coloured arrow; the time-course of the concentration of AZM in the urine are shown in addition to that of bicarbonate and sodium ions as well as of the urinary pH. AA, amino acid; AQP, aquaporin; GM, glomerular membrane; NHE_3_, sodium hydrogen exchanger; kNBC1, sodium bicarbonate transporter. It is assumed that CO_2_ is transferred by aquaporin and protein gas channels ^98^. The thickness of arrows indicate the status of the activity of carbonic anhydrase and the transporters. In case of ingestion of a sustained (retard) AZM, the maximal concentrations may be different and longer lasting ^11–13^. For more details, see the text.

AZM is the prototype of a class of agents that have limited usefulness as diuretics but have played a major role in th development of fundamental concepts of renal physiology and pharmacology^90^. Davenport and Wilhelmi were the first to discover carbonic anhydrase in the mammalian kidney^90^. AZM is a strong inhibitor of the isozymes hCA II and hCA IV^5^. Inhibition of the intrinsic carbonic anhydrase activity of the cytosolic hCA II and the membrane-bound hCA IV in the proximal tubule cells of the nephron results in inhibition of the reabsoprion of filtered HCO_3_^–^ (Figure 3). Pharmacological AZM results in a manifold increase of the HCO_3_^–^ concentration in the urine and in an increase of the urinary pH value up to values of about 8.0, which is the close to the upper physiological urinary pH range (4.5–8.5)^91^. These effects are accompanied by considerable increases in the excretion of Na^+^, K^+^, Cl^–^, H_2_PO_4_^–^ and in a decrease of the NH_4_^+^ excretion^90^.

In blood, the HCO_3_^–^ concentration and the pH value decrease as well, yet to much smaller degrees compared to urine. Ingested AZM is rapidly and nearly completely aborbed and is thoroughly distributed in the blood and in tissues including the proximal tubule and reaches µM-concentrations. Pharmacological AZM is excreted and in part renally secreted in the urine almost unchanged at concentrations in the low µM-range. Circulating and urinary concentrations of a single AZM dose (e.g. 5 mg/kg) are closely associated with each other and ensure AZM concentrations that should sufficy for maximal inhibition of renal hCA II and hCA IV activity for several hours in terms of urinary HCO_3_^–^ concentration and urinary pH value. While this is true for urinary pH values that remain within a slightly alkaline region (pH range, 7–8) for several hours, the concentration of HCO_3_^–^ in the urine reaches maximum concentrations after about 2 h. Peak urinary HCO_3_^–^ concentrations are suddenly reached and are relatively short-lasting for regular tablets and longer-lasting for sustained (retard) AZM tablets at similar dosages. Subsequently, urinary HCO_3_^–^ concentrations drop first abruptly and then slowly to levels that are higher than the baseline values for several hours. In this regime, there is no close association between urinary HCO_3_^–^ concentration and urinary pH (Figure 3).

Oral administration of AZM is accompanied by considerable changes in the concentrations of inorganic cations and anions (including Na^+^, K^+^, NH_4_^+^, Cl^–^) and neutral substances such as H_2_O and gaseous CO_2_. The changes of the above mentioned are much stronger in the urine compared to blood. For instance, the meta-analysis of clinical trials by Schmickl and colleagues resulted in mean differences between AZM and placebo administration of −0.07 units for pH, −2.8 mmHg for pCO_2_, +4.9 mmHg for pO_2_, − 4.5 mM for bicarbonate, +3.3 mM for Cl^–^, ±0 mM for Na^+^, −0.3 mM for K^+^ in blood^75^. AZM administration is also associated with changes in the concentrations of other inorganic anions such as nitrite and nitrate, and of organic substances such as physiological amino acids, which are zwitterionic at physiological pH values of the blood (Figure 3). Other drugs such as salicylate^55^^,^^56^, which is an organic anion, and furosemide^71^ that is a diuretic and saluteric drug primarily acting on the Henle’s loop of the nephron^90^, are known to affect the key pharmacological effects of AZM, albeit by different mechanisms. One primarily transpont independent mechanism could be reduction of the protein binding of AZM that increases its free concentration required for inhibition of carbonic anhydrase activity. Research reporting on the above mentioned “side-effects” of AZM suggests that the inhibition of hCA II and hCA IV in the proximal tubule of the nephron is for the most part due to physiological responses of the nephron on the acute, great and transient changes in the concentration of the HCO_3_^–^/CO_2_ system and the long-lasting, sligthly alkaline pH of the urine. Acute “side-effects” of AZM are likely to concern the activity of transport systems that are abundantly present in the nephron including the Na^+^/H^+^ exchanger, the Na^+^/HCO_3_^–^ co-transporter, and AQP1^68–70^. In addition to these “indirect” affects, AZM seems to exert “direct” effects on renal transporters by inhibiting or elevating their physiological activity. AZM significantly decreases NHE3 activity^42^, inhibits osmotic water permeability by interacting with AQP1^45^^,^^46^, possibly explaining the diuretic effect of AZM. In addition, AZM can affect the activity of renal enzymes including glutamyltransferase^22^, alpha-ketoglutarate dehydrogenase and succinyl-CoA synthetase^62^, possibly contributing to the metabolic and energetic homeostasis of the proximal tubule. Changes in intra-cellular HCO_3_^–^ concentrations in the proximal tubule may be associated with changes in the activity of other enzymes such as the bicarbonate-stimulated soluble adenylyl cyclase^92^ and guanylyl cyclases^93^. Soluble adenylyl cyclase generates the second messanger cyclic adenosine monophosphate (cAMP, 3′,5′-cyclic adenosine monophosphate, and is considered to play a role in acid-base sensing in the kidney^92^. Given the long elimination half-life of AZM of 6–9 h^90^ and its relatively high intra- and extra-cellular µM-concentrations that may prevail for several hours even after administration of a single regular dose (e.g. 5 mg/kg) (Figure 3(B)), it is possible that many “side-effects” of AZM are in part indepedent of its inhibitory effect of carbonic anhydrase activity. However, there is no convincing evidence that AZM may exert changes in the metabolism of cyclic AMP in rat kidney^94^. Also AZM and parathyroid hormone (PTH) were found not to influence the phosphorylation of carbonic anhydrase^94^.

Figure 3(B) shows schematically the temporal pharmacokinetic of AZM concentration in the urine and some of its pharmacodynamic effects on the proximal tubule and after administration of a single oral dose of AZM. The effects on and in proximal tubule cells are multiple. They are not strictly restricted to the primary inhibitory action on the intrinsic activity of hCA II and hCA IC, i.e. the hydration of CO_2_ and the protonation of HCO_3_^–^. The interruption of the physiological function of carbonic anhydrase by AZM in the renal proximal cells is associated with temporal and spatial phenomena along the entire nephron. Because of the ubiquity of carbonic anhydrase, analogous phenomena are likely to occur in other organs including eyes and brain. The abrupt AZM-induced changes in the acid-base balance in the proximal convoluted tubule has immediate effects on other parts of the nephron, including the loop of Henle and the collecting tubule, of which the resorption efficacy depends on the efficacy of the proximal tubule.

Despite sufficiently high concentrations for complete inhibition of the activity of hCA II and hCA IV in proximal tubule cells, its inhibitory effects are incomplete. The pictures shown in Figure 3 suggest that AZM exerts its highest effect on bicarbonate excretion immediately after its complete absorption, which is associated with its maximum excretion in the urine. Seemingly, the bicarbonate excretion is dissociated from the alkanization of the urine, which remains high over several hours, unlike to bicarbonate excretion. Bicarbonate concentrations of a few mM seem to be sufficient to keep the pH value of the urine close to 8. One explanation for the apparently extraordinary pharmacodynamic behavior of AZM could be that the inhibitory action of AZM on hCA II and hCA IV in proximal tubule cells decreases with time for unknown reasons. The concentration of AZM in the blood and in the cytosol of proximal tubule cells is expected to be much higher than its concentration in the primary urine. On the other hand, lack of proteins in the primary urine would be associated with a higher inhibitory potency/efficacy of AZM, while the largest fraction of intracellular AZM would be bound to proteins, thus leading to a diminished inhibitory potency of AZM towards to the cytosolic hCA II. The same would also appy to basolateral hCA IV. In such a scenario, the falling effect of AZM on the excretion of bicarbonate after its maximum effects could be due to dropping concentrations in the primary urine. While the cooperation between hCA II and hCA IV is perfect in the proximal tubule cells of healthy subjects under physiological conditions, AZM seems to acutely and sustainably disturb their closely coordinated action with each other, as well as with resorptions mechanisms for low-molecular-mass physiological substances notably including electrolytes and electrolytes.

## Tolerance development to acetazolamide

Tolerance development to organic nitrates, notably nitroglycerine, is perhaps the oldest and still unexplored phenomenon for tolerance development to pharmacological drugs^23^. Unlike organic nitrates, AZM obviously does not require bioactivation in order to inhibit carbonic anhydrase. Phase-II metabolism of AZM seems to be negligible and, moreover, it is rather unlikely that the glucuronide and acetylated metabolites of AZM are inhibitors of the activity of renal hCA II and h CA IV and of other enzymes in humans.

With respect to aspirin (acetylsalicylic acid) used in the secondary prevention of cardiovascular disease, a considerable portion of patients do not respond appropriately to aspirin. This phenomenon is known as aspirin resistance rather than as aspirin tolerance^95^.

Proposed factors and mechanisms for aspirin resistance include poor medication adherence, high platelet turnover due to underlying inflammatory processes, such as atherosclerosis and its complications, leading to faster regeneration of platelets, identification of platelet glycoprotein IIIa, and the discovery of an anion efflux pump that expels intracellular aspirin from platelets^95^. The case of AZM seems to behave distinctly different from acetylsalicylic acid and organic nitrates.

Prompt loss of diuretic action during continued administration of the AZM has been reported very early in the clinical use of AZM^24^^,^^25^. Intervals of one and two days between single doses failed to restore the initial response level^25^. Rather, 5 to 6 days are required for full recovery of a series of AZM doses, and 2 to 4 days for recovery from an initial single dose^25^. A kind of metabolic acidosis was proposed as a possible mechanism^25^.

Explanations for the development of tolerance to AZM have been proposed and include indirect and direct actions of the drug with kidney transporters including AQP1 as outlined above^45–47^^,^^87^^,^^88^. The study by Martens and colleagues showed that patients with higher circulating baseline bicarbonate concentrations have a higher improvement of decongestive response^71^. On the other hand, higher bicarbonate concentrations in the urine may occur for several hours after oral administration of AZM compared to baseline. The activity of luminal hCA IV could be inhibited by the higher biocarbonate concentrations^18^ that resulted from the preceding AZM dose, thus decreasing the diuretic and saluretic effect of the drug.

In vivo, for instance in the proximal tubule cells of the nephron upon AZM administration, the conditions prevailing for hCA II and hCA IV may be distinctly different from those used *in vitro*, for example when measuring the hydratase activity of carbonic anhydrase using saturated solutions of gasous CO_2_ or CO_2_ generated from added NaHCO_3_^18^^,^^52^^,^^96–98^. Sulphonamides including AZM have been proposed to bind with high affinity to Zn^2+^ of CA II as an anion, i.e. R-SO_2_NH**^–^**^0.5^^96^^,^^99–103^. Because of the lack of appreciable metabolism of AZM, such effects are unlikely to occur in the case of pharmacological AZM and to explain drug tolerance/resistance to this drug.

## Carbonic anhydrase activities beyond CO_2_ hydration

Carbonic anhydrase also catalyse the reaction of water with other electrophiles such as aromatic esters, sulphates and phosphates, thus lending to carbonic anhydrase esterase, sulfatase and phosphatase activity, respectively^104^. These reactions are independent of CO^2^, yet it should be emphasised that CO_2_ and bicarbonate are ubiquitous in biological systems. Human and bovine CA II and CA IV also possess nitrous acid anhydrase activity^15^, which can be enhanced by bicarbonate at physiological concentrations, but not inhibited by AZM *in vitro*^97^^,^^105^. Carbonic anhydrase activity can be inhibited by several types of drugs indicating the existence of several inhibition mechanisms^106^. Reversely, the multiple pharmacological side-effects of AZM^75^ suggest that AZM may interfer with the activity of other enzymes despite its high affinity to hCA II and hCA IV, as has been observed for other sulphonamides^57^, and as has been discussed above. With other words, AZM seems to lack pharmacological specificity and many of its actions, such as the local vasodilatory effect on cerebral arterioles in humans^107^, seem to be unrelated to its specific effects as a carbonic anhydrase inhibitor and include direct activation of calcium-activated potassium (K(Ca)) channels^108^.

## Conclusion

In conclusion, carbonic anhydrase is one of the best investigated family of enzymes. Carbonic anhydrase catalyses very effectively a very simple, yet indispensable to life chemical reaction. Carbonic anhydrase is the main pharmacological target of AZM, a simply structured sulphonamide. AZM has high affinity to hCA II and hCA IV. Its acute action in the proximal tubule of the nephron leads to strongly elevated, yet short-lasting excretion of HCO_3_^–^ and Na^+^ in the urine and much weaker effects in the blood except for acidosis. The long-lasting concentrations of AZM in blood and urine do not translate into long-lasting pharmacodynamic effects, and suggest loss of potency even upon single administration. AZM is therapeutically used in many conditions and exerts numerous side-effects suggesting additional effects independent of carbonic anhydrase inhibition. In the kidney, AZM seems to influence directly and indirectly the activity of transporters of electrolytes, amino acids, and water, and of many metabolic enzymes. In chronic use, AZM rapidly drops pharmacological activity resembling the development of tolerance to nitroglycerine and drug resistance to aspirin, although AZM is a reversible inhibitor, at least of carbonic anhydrase activity, and seems not to require preceding bioactivation as far we know up to date, after almost eight decades of research on various aspects of carbonic anhydrase including searching for both, inhibitors and activators of this enzyme family^109^. The recently discovered nitrous anhydrase activity of hCA II and hCA IV is not inhibitable by AZM, suggesting distingly different, HCO_3_^–^/CO^2^ independent mechanism^105^. Finally, it cannot be excluded that other proteins such as ubiquitin possess weakly AZM-inhibitable CO_2_ hydratase activity^110^.

Furosemide is another sulphonamide type strong inhibitor of hCA II (*K*_i_^65^, nM), but not of hCA IV (*K*_i_, 564 nM)^111^. In a direct comparison of AZM (50 mg bolus i.v.) and furosemide (40 mg bolus i.v.) in critically ill patients^112^, furosemide increased about 5-fold the urinary excretion of Na^+^ and Cl^–^ compared to AZM. These observations suggest that that combined use of AZM and furosemide may achieve furosemide-associated diuresis, natriuresis, chloriuresis without the acid-base side effects of AZM when used alone^112^. Classically, diuretics are classified according to their chemical structure, their mechanism and primary site of action within the nephron, and their diuretic potency^6^. However, diuretics have several sites of action. AZM can also act on the collecting duct and inhibit the luminal Na^+^/K^+^/2Cl^–^ tranporter in the luminal cell membrane of the ascending limb of the loop of Henle, whereas furosemide can inhibit the activity of hCA II and hCA IV in the proximal tubule cells^6^.
